# Prevalence and associated factors of waterpipe tobacco smoking in Japan

**DOI:** 10.18332/tid/205196

**Published:** 2025-07-12

**Authors:** Yukari Isaka, Ai Hori, Bibha Dhungel, Takahiro Tabuchi, Kota Katanoda

**Affiliations:** 1 Institute of Medicine, University of Tsukuba, Tsukuba, Japan; 2 Institute of Medicine, Transborder Medical Research Center, University of Tsukuba, Tsukuba, Japan; 3 Population Interventions Unit, Melbourne School of Population and Global Health, The University of Melbourne, Parkville, Australia; 4 Department of Health Policy, National Center for Child Health and Development, Setagaya, Japan; 5 Division of Epidemiology, School of Public Health, Graduate School of Medicine, Tohoku University, Sendai, Japan; 6 Division of Population Data Science, National Cancer Center Institute for Cancer Control, Tokyo, Japan

**Keywords:** tobacco, smoking, hookah, waterpipe, shisha

## Abstract

**INTRODUCTION:**

Despite the global increase in waterpipe tobacco use (hookah tobacco, shisha), its prevalence and characteristics are understudied in Japan. We aimed to investigate the prevalence and associated factors of waterpipe tobacco use in the Japanese population.

**METHODS:**

Data were obtained from the Japan ‘Society and New Tobacco’ Internet Survey, conducted in February 2023. Of the 34000 surveyed participants, a total of 31037 respondents (91.3%) aged 15–82 years were included. Current waterpipe tobacco smokers were defined as those reporting use ‘occasionally’ or ‘almost every day’ in the past 30 days. Inverse probability weighting was used to calculate waterpipe tobacco use, which approximated the Japanese national population. Logistic regression analyses were conducted to estimate the effect of waterpipe tobacco use, adjusted for sociodemographic variables.

**RESULTS:**

The prevalence of current and former waterpipe tobacco use in Japan was 1.4% and 3.9%, respectively, both with higher prevalence among men. Use was notably higher among individuals in their 20s, with prevalence rates of 4.4% among those aged 20–29 years. Waterpipe tobacco use among current tobacco product users was 2.5% for cigarette users, 4.7% for heated tobacco product users, and 32.5% for electronic cigarette users. Waterpipe tobacco use was more strongly associated with current e-cigarette (AOR=4.06; 95 % CI: 2.7–6.0) and heated tobacco product (HTP) use (AOR=2.44; 95 % CI: 1.9–3.2), while individuals with former combustible tobacco (AOR=3.12; 95 % CI: 2.4–4.1) or cannabis use (AOR=14.8; 95 % CI: 9.5–23.2) reported higher prevalence of current waterpipe use.

**CONCLUSIONS:**

We found a high prevalence of waterpipe tobacco smoking among young adults and other tobacco product users in Japan. Public health initiatives should focus on educating the population about the risks associated with waterpipe tobacco use and developing policies to regulate its availability and marketing.

## INTRODUCTION

Waterpipe tobacco, also known as hookah tobacco or shisha, is smoked using a method in which heated tobacco smoke is inhaled through water using a device called a waterpipe. In recent years, the prevalence of waterpipe tobacco use has been increasing worldwide, particularly among young adults^1^. In a 2013 survey conducted among individuals aged 18–24 years in the United States, the prevalence of waterpipe tobacco use was 28.4%, surpassing the 19.6% rate of cigarette use^2,3^. Another study showed that approximately half of waterpipe tobacco users are non-smokers, and waterpipe tobacco use can serve as an introduction to the use of other tobacco products^4^. These findings suggest that the use of waterpipe tobacco not only increases health risks and nicotine addiction but might also be a gateway to the use of other tobacco products^5^ .

Smoking of waterpipe tobacco mainly occurs in cafes and bars, where the use patterns may differ from those of other tobacco products. According to a web-based survey conducted in Japan during 2022 by a private company, the number of shisha bars or shisha cafes where waterpipe tobacco is available is increasing in Japan, as in other countries^6^. A previous study showed that a higher tobacco retailer density near the home of adolescents predicts greater odds of their initiating any alternative tobacco product use^7^. Therefore, also the rapid increase in the number of shisha bars or shisha cafes in Japan might contribute to a rise in the number of Japanese waterpipe tobacco users.

Although the rates of combustible cigarette smoking have seen a decreasing trend over the past decade in Japan, the spread of waterpipe tobacco use may lead to a rise in alternative tobacco product smoking and the introduction of non-smokers to these tobacco products. Whereas other countries are beginning to evaluate the use of waterpipe tobacco and its impact on other tobacco products, no such studies have been conducted in Japan. Therefore, the objective of this study is to estimate the prevalence and associated factors of waterpipe tobacco use in the Japanese population.

## METHODS

### Study design and sample

We obtained data from the Japan Society and New Tobacco Internet Survey (JASTIS), a longitudinal survey randomly sampling members of the survey panel provided by Rakuten Insight, a major internet research group in Japan. The survey was conducted between 6 and 27 February 2023. Further details are available in a previous report^8^. Of the 34000 study participants, we excluded respondents with discrepancies or invalid responses (n=2963) from our analyses. Finally, 31037 participants were included in the analysis.

### Measures

*Tobacco product use*

We included data from all respondents to the 2023 survey. Waterpipe tobacco use was defined as use in the past 30 days. Other tobacco product use was determined with the following question: ‘Do you currently use any of the following tobacco products? (cigarettes, roll-your-own cigarettes, Ploom Tech, Ploom S, Ploom X, IQOS, Glo, lil HYBRID, electronic cigarettes containing nicotine, electronic cigarettes without nicotine, electronic cigarettes with unknown nicotine content, and waterpipe tobacco)’. The response options were ‘never’, ‘several times in the past’, ‘habitually in the past’, ‘occasionally’, and ‘almost every day’. We defined those who answered ‘occasionally’ or ‘almost every day’ as current smokers, ‘habitually in the past’ or ‘several times in the past’ as ‘former smoker’, and ‘never’ as never smokers.

*Other variables*

The factors measured included age group (15–19, 20–29, 30–39 or 40–49, 50–59, and ≥60 years), sex (male or female), educational status (high school or less, college or more), equivalent household income in million JPY (1000 Japanese Yen about US$7) (1st quartile, ≤2.25; 2nd quartile, 2.26–3.25; 3rd quartile, 3.26–4.75; 4th quartile, ≥4.76; unknown/declined to answer), alcohol use (current, former, have not drunk in the past year, never), cannabis use (current, former, never), residence district (densely inhabited district [DID]: metropolitan area, large city, accessible small town, remote small town, accessible rural settlement, remote rural settlement), and residential prefecture. We used DID data, based on the 2015 Population Census of Japan, described elsewhere^9^.

### Statistical analysis

We calculated unweighted and weighted percentages of waterpipe tobacco product use among all survey respondents according to each variable. We applied inverse probability weighting^8^ for weighted results to account for differences between the sociodemographic characteristics of survey respondents and those of the Japanese general population. Data from JASTIS and a probability sample from the Comprehensive Survey of Living Conditions of People on Health and Welfare^10^ were combined and used in a logistic regression model with covariates to estimate the probability of being an internet survey respondent, i.e. the propensity score. In accordance with a previous study, the variables included in the propensity score model were area of residence, education level, marital status, housing tenure, occupation, health-rated health, and smoking status^11^.

We then conducted unadjusted logistic regression analyses to estimate the association of waterpipe tobacco use and each variable with odds ratios (ORs) and its 95% confidence intervals (CIs). Finally, we conducted multiple logistic regression analyses to estimate the association of waterpipe tobacco use, controlling for age, sex, education level, equivalent household income, alcohol use, smoking status (combustible tobacco use, HTP use, e-cigarette use), cannabis use, and DID, with adjusted odds ratios (AORs) and 95% confidence intervals (CIs). All analyses were conducted using Stata SE version 18.0 (StataCorp LLC, College Station, TX, USA). All statistical tests were twosided, and a significance level of 0.05 was used.

### Ethical approval

The study protocol and informed consent procedure were reviewed and approved by the Research Ethics Committee of the Osaka International Cancer Institute (no. 20084).

## RESULTS

Table 1 shows characteristics of the study participants. Upon survey, 1.4% (95% CI: 1.2–1.6) of respondents were current waterpipe tobacco users, and 3.9% (95% CI: 3.6–4.2) were former users.

**Table 1:** Characteristics of study participants, JASTIS, February 2023 (N=31037)

*Characteristics*	*Unweighted* *%*	*Weighted ^a^*		
		*n*	*%*	*95% CI*
**Sex**				
Male	49.4	15343	49.4	48.6–50.3
Female	50.6	15694	50.6	49.7–51.4
**Age** (years)				
15–19	2.3	722	2.3	2.1–2.6
20–29	17.7	5487	17.7	17.0–18.4
30–39	18.5	5752	18.5	17.9–19.2
40–49	17.7	5485	17.7	17.1–18.3
50–59	14.7	4573	14.7	14.2–15.3
≥60	29.1	9018	29.1	28.2–29.9
**Education level**				
High school, technical school	39.1	19980	65.3	64.6–66.0
College, university or higher	60.9	10610	34.7	34.0–35.1
**Equivalent household income** (million JPY)				
1st quartile (≤2.25)	18.8	6570	21.2	20.4–21.9
2nd quartile (2.26–3.25)	19.6	6380	20.6	19.9–21.3
3rd quartile (3.26–4.75)	19.6	5463	17.6	17.0–18.2
4th quartile (≥4.76)	20.4	5139	16.6	16.0–17.1
Unknown/declined to answer	21.7	7485	24.1	23.4–24.9
**Alcohol use**				
Never	33.8	10968	35.3	34.5–36.2
Former	4.4	1472	0.48	0.4–0.5
Current	61.8	18597	59.9	59.1–60.8
**Smoking status** (last 30 days)				
**Waterpipe tobacco**				
Never user	94.0	29390	94.7	94.3–95.1
Current user	1.4	438	1.4	1.2–1.6
Former user	4.6	1209	3.9	3.6–4.2
**Combustible tobacco**				
Never user	83.3	25392	81.8	81.1–82.5
Current user	9.3	3114	10.0	9.5–10.6
Former user	7.5	2531	8.2	7.7–8.6
**HTPs^b^**				
Never user	89.1	27383	88.2	87.7–88.8
Current user	7.5	2582	8.3	7.9–8.8
Former user	3.4	1073	3.4	3.2–3.8
**E-cigarettes^c^**				
Never user	96.9	30037	96.6	96.5–97.1
Current user	1.2	400	1.3	1.1–1.5
Former user	1.9	599	1.9	1.7–2.2
**Cannabis use**				
Never user	95.7	29724	95.8	95.4–96.1
Current user	0.9	254	0.8	0.7–1.0
Former user	3.4	1059	3.4	3.1–3.8
**Densely Inhabited District** (DID)				
Metropolitan area	49.3	10614	35.1	34.3–35.8
Large city	15.4	5534	18.3	17.6–19.0
Accessible small town	5.7	1881	6.2	5.8–6.7
Remote small town	5.3	2040	6.7	6.3–7.2
Accessible rural settlement	13.3	5082	16.8	16.1–17.5
Remote rural settlement	11.0	5122	16.9	16.2–17.7

a Adjusted for ‘being a respondent in an internet survey’ using a nationally representative sample in Japan.

b Heated tobacco products (Ploom, IQOS, Glo and lil HYBRID).

c Electronic cigarettes (nicotine e-cigarettes, non-nicotine e-cigarettes, e-cigarettes with unknown nicotine).

JPY: 1000 Japanese Yen about US$7.

Supplementary file Table S1 shows that waterpipe tobacco use varied according to participants’ demographic characteristics. Approximately three-fourths of waterpipe tobacco users (2.1%; n=331) were male individuals. Notably, young adults aged 20–29 years had the highest prevalence, both overall (4.4%; 95% CI: 3.5–5.4) and by sex; this was followed by adolescents with 2.0% (95% CI: 1.1–3.7). A higher level of education (2.4%; 95% CI: 2.0–2.8) was marginally associated with higher waterpipe tobacco use than a lower level of education (2.0%; 95% CI: 1.5–2.6) in male respondents; there was no difference in female respondents (0.7%). Higher income levels had higher rates of waterpipe tobacco use in female respondents, but this tendency was reversed in male respondents. Only 2.5% and 4.7% of current cigarette or heated tobacco product (HTP) users, respectively, currently used waterpipe tobacco; however, 32.5% (95% CI: 25.1–40.8) of current electronic cigarette (e-cigarette) users currently used waterpipe tobacco. Among cannabis users, 34.5% (95% CI: 25.9–44.3) were also currently using waterpipe tobacco. Similarly, former users of cigarettes (9.9%; 95% CI: 8.3–11.9), HTPs (20.9%; 95% CI: 17.2–25.1), and e-cigarettes (29.5%; 95% CI: 24.3–35.2) were currently using waterpipe tobacco. Additionally, living in a metropolitan area (1.7%; 95% CI: 1.4–2.2) was associated with greater waterpipe tobacco use than living in other residential areas.

**Figure 1:**
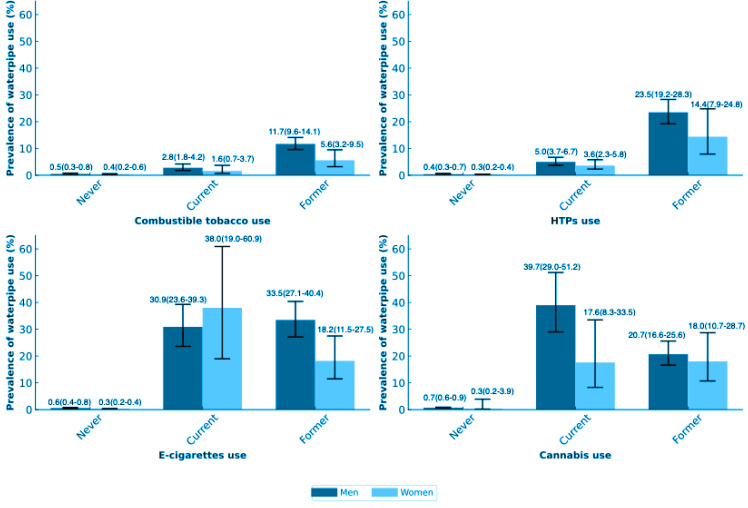
Weighted prevalence of waterpipe tobacco use in Japan by smoking status and cannabis use, separately by sex

Figure 1 shows that the weighted prevalence of waterpipe use was higher among female current e-cigarette users (38.0%; 95% CI: 19.0–60.9) than among males (30.9%; 95% CI: 23.6–39.3). Table 2 shows the association of current use of waterpipe tobacco and each variable. Waterpipe tobacco use was related to male sex (AOR=1.30; 95% CI: 1.1–1.6), higher level of education (AOR=1.20; 95% CI: 1.0–1.4), higher income category (AOR=1.59; 95% CI: 1.2–2.1), current alcohol use (AOR=1.91; 95% CI: 1.5–2.4), living in a metropolitan area (AOR=1.74; 95% CI: 1.3–2.4), and younger age. As for the relative effect of using other tobacco products and cannabis, waterpipe tobacco use was more prevalent among respondents with current e-cigarette use (AOR=4.06; 95% CI: 2.7–6.0) than in those with former e-cigarette use (AOR=2.98; 95% CI: 1.9–4.7). Waterpipe tobacco use was also more common among respondents with current HTP use (AOR=2.44; 95% CI: 1.9–3.2) than in those with former use (AOR=2.26; 95% CI: 1.6–3.2). However, respondents with former combustible tobacco use (AOR=3.12; 95% CI: 2.4–4.1) and former cannabis use (AOR=14.8; 95% CI: 9.5–23.2) smoked waterpipe tobacco more often than those with current use.

**Table 2:** Current use of waterpipe tobacco products among participants aged 15–82 years

*Variables*	*Waterpipe tobacco use ^a^*			
	*OR*	*95% CI*	*AOR ^b^*	*95% CI*
**Sex**				
Male	2.67	2.3–3.1	1.30	1.1–1.6
Female ®	1		1	
**Age** (years)				
15–19	4.52	2.5–8.1	4.47	2.6–7.8
20–29	9.86	6.9–14.2	5.60	3.9–8.2
30–39	6.51	4.5–9.4	4.16	2.8–6.1
40–49	4.44	3.1–6.5	2.74	1.8–4.0
50–59	1.97	1.3–3.0	1.44	0.9–2.2
≥60 ®	1		1	
**Education level**				
High school, technical school ®	1		1	
College, university or higher	1.37	1.2–1.6	1.20	1.0–1.4
**Equivalent household income** (million JPY)				
1st quartile (≤2.25) ®	1		1	
2nd quartile (2.26–3.25)	0.94	0.7–1.2	1.00	0.7–1.4
3rd quartile (3.26–4.75)	1.31	1.0–1.6	1.08	0.8–1.4
4th quartile (≥4.76)	1.91	1.5–2.4	1.59	1.2–2.1
Unknown/declined to answer	0.55	0.4–0.7	0.76	0.6–1.0
**Alcohol use**				
Never ®	1		1	
Former	1.36	0.9–2.1	1.23	0.8–1.9
Current	2.60	2.2–3.1	1.91	1.5–2.4
**Smoking status** (last 30 days)				
**Combustible tobacco**				
Never user ®	1		1	
Current user	1.90	1.5–2.4	0.99	0.7–1.3
Former user	10.20	8.6–12.1	3.12	2.4–4.1
**HTPs^c^**				
Never user ®	1		1	
Current user	5.93	4.9–7.1	2.44	1.9–3.2
Former user	22.00	17.7–27.2	2.26	1.6–3.2
**E-cigarettes^d^**				
Never user ®	1		1	
Current user	22.71	16.5–31.3	4.06	2.7–6.0
Former user	30.55	23.5–39.7	2.98	1.9–4.7
**Cannabis use**				
Never user ®	1		1	
Current user	50.36	34.0–74.7	7.87	6.0–10.4
Former user	21.00	17.0–26.0	14.83	9.5–23.2
**Densely inhabited district** (DID)				
Metropolitan area	2.13	1.62–2.79	1.74	1.3–2.4
Large city	1.21	0.87–1.67	1.21	0.8–1.8
Accessible small town	1.21	0.75–1.93	1.23	0.8–2.0
Remote small town	1.25	0.82–1.89	1.38	0.9–2.2
Accessible rural settlement	1.00	0.72–1.41	0.95	0.6–1.4
Remote rural settlement ®	1		1	

a Use in the last 30 days.

b AOR: adjusted odds ratio; adjusted for sex, age group, education level, equivalent household income, alcohol use, smoking status (combustible tobacco use, HTP use, e-cigarette use), cannabis use, and DID.

c Heated tobacco products (Ploom, IQOS, Glo and lil HYBRID).

d Electronic cigarettes (nicotine e-cigarettes, non-nicotine e-cigarettes, e-cigarettes with unknown nicotine).

JPY: 1000 Japanese Yen about US$7. ® Reference categories.

## DISCUSSION

This study estimated the prevalence and associated factors of waterpipe tobacco use in Japan. The results revealed a high prevalence of waterpipe use, particularly among individuals in their 20s, and indicated a higher prevalence of concurrent use of combustible tobacco, HTPs, e-cigarettes, and cannabis.

The overall prevalence of waterpipe tobacco use in Japan is 1.4%. This was associated with the age group of 20–29 years (AOR=5.60; 95% CI: 3.9–8.2), in comparison with other age groups, with a notable disparity between sexes (AOR=1.30; 95% CI: 1.1–1.6). The association with waterpipe use of male sex, younger age, higher socioeconomic status, and urban residence in our study matched previous reports^1^. The discrepancy in the prevalence of waterpipe use in Japan compared with other countries might be attributed to fewer establishments offering waterpipe tobacco in Japan than in other countries. The age-related pattern raises questions about the factors driving waterpipe tobacco consumption among young adults, including easy access, social influences, marketing strategies, or perceptions of waterpipe tobacco as a fashionable or trendy activity^12,13^.

Currently, only 1.4% of Japanese residents use waterpipe tobacco. The prevalence of shisha use was highly associated with the concurrent use of cigarettes, HTPs, and with e-cigarettes in particular (AOR=4.06; 95% CI: 2.7–6.0). As in previous studies, waterpipe tobacco users reported high rates of concurrently using flavored tobacco products, cannabis, and other tobacco products^14,15^. The present survey results highlight the interconnectedness of substance use behaviors.

Previous studies show that flavored tobacco has a substantial effect on enhancing the waterpipe smoking experience. Also, waterpipe tobacco is perceived as being safer than cigarettes or HTPs. These factors mainly account for the increased use of waterpipe smoking^16-18^. Moreover, owing to differences in frequency and quantity of use, caution is necessary when comparing waterpipe tobacco use with other tobacco products. Although waterpipe smoking may be intermittent, the intake of waterpipe tobacco during one smoking session of use may result in greater intake of carbon monoxide and a dramatically greater amount of smoke than use of other products^19^. Considering the potential impact of waterpipe tobacco use patterns on the likelihood of initiating use of other substances or alternative tobacco products, as well as poor knowledge and awareness about the harm of waterpipe tobacco use among the Japanese population, further research is needed.

In recent years the number of waterpipe tobacco shops in Japan has experienced rapid growth, expanding from approximately 10 establishments in 2018 to 1013 in 2023, with the market size expanding annually. Furthermore, there is at least one establishment registered for smoking purposes in each of the 47 prefectures of Japan. Additionally, previous research suggests that tobacco outlets densely located around the residences of young people and students may have an environmental impact on the use of tobacco products by young people^7^. The distribution of waterpipe tobacco use across prefectures and its relationship with the number of waterpipe tobacco stores have potential regulatory and policy implications. Regions with a higher prevalence of waterpipe tobacco use may require stricter regulations on the establishment and operation of waterpipe tobacco stores to mitigate potential public health risks associated with increased consumption. Future study is necessary to ascertain the actual situation regarding shisha bars in residential areas of each city and to clarify the proximity to people and these establishments.

### Strengths and limitations

The main strength of this study is that we used a nationwide sampling database established after waterpipe smoking had become popular in Japan and that includes demographic and weighted data to ensure representativeness. Furthermore, by querying waterpipe users regarding the use of other tobacco products and substances (combustible cigarettes, e-cigarettes, HTPs, cannabis use), we revealed that waterpipe users may be multi-substance users. However, this study has several limitations. First, we applied a cross-sectional design, which limits our ability to establish causality or determine temporal relationships. However, we aimed to the estimate the prevalence of waterpipe tobacco use in the Japanese population and its association with the use of other tobacco products. In the case of multi-substance use, the temporal relationship between the use of waterpipe tobacco products and other tobacco products or cannabis is unknown and should be investigated using longitudinal analysis. Second, our findings are based on data obtained through questionnaire surveys, which may be subject to recall bias and social desirability bias. However, we adjusted to account for possible bias in the collected sample using an external, nationally representative sample. Furthermore, the use prevalence for each tobacco product in this study approximated other Japanese national data^20^. Third, we did not ascertain whether the waterpipe tobacco used by survey respondents contained nicotine. This lack of information hinders comprehensive understanding of waterpipe tobacco use patterns. Future studies should include measures to accurately determine the nicotine content of waterpipe tobacco products, which is crucial for assessing health risks and informing public health interventions. Acknowledging these limitations is essential to accurately interpret the findings of this study and will inform future research efforts to address any identified gaps.

## CONCLUSIONS

The study provides a comprehensive overview of current waterpipe tobacco use in Japan, which was associated with younger populations and users of other tobacco products, especially e-cigarettes. Considering the characteristics of waterpipe tobacco users, use patterns, and product features, ongoing longitudinal research is needed regarding the association with other products, as well as specialized surveys focusing on identifying common times and locations of waterpipe tobacco use.

## Supplementary Material



## Data Availability

The data can be accessed via the principal investigator of the Japan “Society and New Tobacco” Internet Survey (JASTIS) study, Takahiro Tabuchi, upon reasonable request. The code used to generate the results presented in the manuscript is available from the corresponding author upon reasonable request.
