# Supplementary material for: Prevalence and associated factors of waterpipe tobacco smoking in Japan
Source: Tob Induc Dis. 2025 Jul 12;23:10.18332/tid/205196. doi: 10.18332/tid/205196 (PMC12254910; doi:10.18332/tid/205196)
Supplement: Supplementary file 1 [file TID-23-94-s1.pdf]

Supplementary file Table S1. Characteristics of waterpipe tobacco users in Japan (JASTIS2023 Feb)

	Waterpipe tobacco use[a]											
Variables	Total				Men				Women			
	Unweighted	Weighted[b]			Unweighted	Weighted			Unweighted	Weighted		
	%	Use	%	95% CI[c]	%	Use	%	95% CI[c]	%	Use	%	95% CI[c]
**Total**	1.4	438/31037	1.4	(1.2-1.6)	2.1	331/15343	2.1	(1.8-2.6)	0.8	107/15694	0.7	(0.5-0.9)
**Age groups (years)**												
15−19	2.6	15/722	2.0	(1.1−3.7)	2.9	7/309	2.1	(0.8−5.8)	2.4	8/413	1.9	(0.9−4.1)
20−29	4.3	240/5487	4.4	(3.5−5.4)	6.3	181/2678	6.8	(5.3−8.5)	2.3	59/2809	2.1	(1.3−3.3)
30−39	1.9	104/5752	1.8	(1.4−2.4)	2.8	86/2820	3.1	(2.3−4.1)	1.0	18/2932	0.6	(0.4−1.0)
40−49	1.0	58/5485	1.1	(0.7−1.6)	1.5	43/2740	1.6	(1.0−2.4)	0.4	15/2745	0.6	(0.2−1.9)
50−59	0.4	12/4573	0.3	(0.1−0.5)	0.5	6/2259	0.3	(0.1−0.5)	0.2	53/2314	0.2	(0.1−0.8)
≥60	0.2	9/9018	0.1	(0.0−0.3)	0.2	7/4537	0.2	(0.0−0.5)	0.1	20/4481	0.1	(0.0−0.2)
**Education level**												
High school, technical school	1.1	263/19980	1.3	(1.0−1.7)	1.8	195/9898	2.0	(1.5−2.6)	0.5	67/10082	0.7	(0.4−1.1)
College, university or above	1.6	160/10610	1.5	(1.3−1.7)	2.1	121/5157	2.4	(2.0−2.8)	1.0	39/5453	0.7	(0.5−0.9)
**Equivalent household income**												
1st quartile (2.25 million JPY and less)	1.9	124/6570	1.9	(1.4−2.5)	3.2	111/2936	3.8	(2.7−5.2)	0.7	14/3634	0.4	(0.2−0.6)
2nd quartile (2.26 to 3.25 million JPY)	1.1	66/6380	1.0	(0.7−1.5)	1.6	55/3207	1.7	(1.1−2.7)	0.5	11/3173	0.3	(0.2−0.7)
3rd quartile (3.26 to 4.75 million JPY)	1.6	86/5463	1.6	(1.2−2.1)	2.1	63/3200	2.0	(1.4−2.8)	1.1	23/2263	1.0	(0.6−1.8)
4th quartile (4.76 million JPY and more)	1.7	103/5139	2.0	(1.5−2.7)	2.2	78/3215	2.4	(1.8−3.3)	1.0	25/1734	1.3	(0.6−2.8)
Unknown/ declined to answer	0.8	58/7485	0.8	(0.5−1.2)	1.1	24/2785	0.9	(0.5−1.4)	0.6	35/4700	0.7	(0.4−1.5)
**Alcohol use**												

Never	0.9	94/10968	0.8	(0.6–1.2)	1.6	71/3953	1.8	(1.2–2.7)	0.5	23/7015	0.3	(0.2–0.6)
Former	1.2	21/1472	1.4	(0.5–3.5)	1.6	6/718	0.9	(1.4–2.1)	0.8	14/753	1.9	(0.5–0.6)
Current	1.7	323/18597	1.7	(1.5–2.1)	2.2	253/10671	2.4	(2.0–2.9)	1.0	69/7926	0.9	(0.6–1.3)
**Smoking status (30 days)[a]**												
**Combustible tobacco**												
Never user	0.4	110/25392	0.4	(0.3–0.6)	0.4	58/11232	0.5	(0.3–0.8)	0.4	54/14161	0.4	(0.2–0.6)
Current user	2.7	77/3114	2.5	(1.7–3.6)	2.7	61/2309	2.8	(1.8–4.2)	2.0	13/805	1.6	(0.7–3.7)
Former user	10.7	251/2531	9.9	(8.3–11.9)	10.6	195/1802	11.7	(9.6–14.1)	7.0	41/729	5.6	(3.2–9.5)
**HTPs[d]**												
Never user	0.4	93/27383	0.3	(0.2–0.5)	0.4	52/12598	0.4	(0.3–0.7)	0.3	41/14785	0.3	(0.2–0.4)
Current user	5.9	121/2582	4.7	(3.6–6.1)	5.7	99/1982	5.0	(3.7–6.7)	6.5	22/600	3.6	(2.3–5.8)
Former user	19.5	224/1073	20.9	(17.2–25.1)	22.7	179/763	23.5	(19.2–28.3)	12.0	45/309	14.4	(7.9–24.8)
**e-cigarettes[e]**												
Never user	0.5	131/30037	0.5	(0.3–0.6)	0.6	85/14586	0.6	(0.4–0.8)	0.4	46/16451	0.3	(0.2–0.4)
Current user	29.6	130/400	32.5	(25.1–40.8)	29.8	97/313	30.9	(23.6–39.3)	28.9	33/87	38.0	(19.0–60.9)
Former user	31.4	177/599	29.5	(24.3–35.2)	33.5	149/444	33.5	(27.1–40.4)	25.5	28/155	18.2	(11.5–27.5)
**Cannabis use**												
Never user	0.5	139/29724	0.5	(0.4–0.6)	0.7	94/14394	0.7	(0.8–0.9)	0.4	42/15317	0.3	(0.2–3.9)
Current user	31.4	89/254	34.5	(25.9–44.3)	35.5	77/195	39.7	(29.0–51.2)	21.6	10/74	17.6	(8.3–33.5)
Former user	18.0	211/1059	19.9	(16.2–24.4)	19.2	156/754	20.7	(16.6–25.6)	15.2	55/303	18.0	(10.7–28.7)
**Densely Inhabited District (DID)**												
Metropolitan areas	1.5	188/10614	1.7	(1.4–2.2)	2.2	158/6260	2.5	(2.0–3.2)	0.8	30/4345	0.7	(0.5–1.0)
Large cities	1.1	52/5534	0.9	(0.7–1.3)	1.7	38/2265	1.7	(1.1–2.5)	0.5	14/3269	0.4	(0.2–0.8)
Accessible small towns	1.1	22/1881	1.1	(0.5–2.8)	1.3	5/641	0.6	(0.3–1.2)	0.9	16/940	1.7	(0.5–5.4)

Remote small towns	1.3	25/2040	1.2	(0.6–2.5)	1.8	19/919	2.1	(0.9–4.8)	0.8	6/1121	0.5	(0.2–1.4)
Accessible rural settlements	1.3	61/5082	1.2	(0.8–1.8)	1.7	42/2323	1.8	(1.1–2.9)	0.8	18/2759	0.7	(0.3–1.3)
Remote rural settlements	1.1	56/5122	1.1	(0.7–1.9)	1.7	42/2211	1.9	(1.1–3.2)	0.5	15/2911	0.5	(0.1–1.9)

[a]Use in the last 30 days; [b]Adjusted for 'being a respondent in an internet survey' using a nationally representative sample in Japan; [c]confidence interval; [d]heated tobacco products (Ploom, IQOS, Glo and lil HYBRID); [e]electronic cigarettes (nicotine e-cigarettes, non-nicotine e-cigarettes, e-cigarettes with unknown nicotine).

© 2025 Isaka Y. et al.
